# Update on the Cognitive Presentations of iNPH for Clinicians

**DOI:** 10.3389/fneur.2022.894617

**Published:** 2022-07-20

**Authors:** Tobias Langheinrich, Cliff Chen, Owen Thomas

**Affiliations:** ^1^Department of Neurology, Manchester Centre for Clinical Neurosciences, Salford Royal NHS Foundation Trust, Salford, United Kingdom; ^2^Division of Neuroscience and Experimental Psychology, School of Biological Sciences, University of Manchester, Manchester, United Kingdom; ^3^Department of Neuropsychology, Manchester Centre for Clinical Neurosciences, Salford Royal NHS Foundation Trust, Salford, United Kingdom; ^4^Department of Neuroradiology, Manchester Centre for Clinical Neurosciences, Salford Royal NHS Foundation Trust, Salford, United Kingdom

**Keywords:** Idiopathic Normal Pressure Hydrocephalus, dementia, Alzheimer's disease, Lewy body disease, progressive supranuclear palsy, corticobasal degeneration, vascular dementia, cognitive

## Abstract

This mini-review focuses on cognitive impairment in iNPH. This symptom is one of the characteristic triad of symptoms in a condition long considered to be the only treatable dementia. We present an update on recent developments in clinical, neuropsychological, neuroimaging and biomarker aspects. Significant advances in our understanding have been made, notably regarding biomarkers, but iNPH remains a difficult diagnosis. Stronger evidence for permanent surgical treatment is emerging but selection for treatment remains challenging, particularly with regards to cognitive presentations. Encouragingly, there has been increasing interest in iNPH, but more research is required to better define the underlying pathology and delineate it from overlapping conditions, in order to inform best practise for the clinician managing the cognitively impaired patient. In the meantime, we strongly encourage a multidisciplinary approach and a structured service pathway to maximise patient benefit.

## Introduction

Idiopathic Normal Pressure Hydrocephalus (iNPH) is a clinical syndrome (1) derived by analogy from NPH but in the absence of a preceding insult (2). iNPH is characterised by the clinical trial of progressive and prominent decline in mobility, followed by less prominent but equally progressive cognitive impairment and bladder disturbance. When supported by ventriculomegaly on brain imaging, the diagnosis should be straightforward. In clinical practise, however, patients suspected of having iNPH frequently present significant cognitive impairment, often preceding or starting at the same time as the mobility disorder. This review focusses on such patients emphasising the clinical, imaging and neuropsychological differential diagnosis, highlighting gaps in our understanding, describing recent developments and making suggestions for future research.

## Clinical

Cognitive impairment in iNPH is not universal but is frequently present. Insidious onset and more prevalent causes of cognitive impairment, such as vascular dementia (VaD) and Alzheimer's disease (AD), make early diagnosis of iNPH challenging. Significant cognitive impairment in the absence of an early, prominent and typical decline in mobility mandates consideration of other underlying causes given their impact on prognosis and therapeutic decision making.

The typical cognitive profile of iNPH is “subcortical” with impaired attention, reduced psychomotor speed and inefficient memory (3).

VaD results in a similar profile, making these two entities difficult to separate on clinical grounds (4). Cerebrovascular disease (CVD) is frequently present on routine MRI scanning but unless severe its significance is often uncertain. Furthermore, neuroimaging findings between iNPH and CVD overlap.

The commonest cognitive profile of AD, that of progressive amnesia (5), may not be detectable early on. Assessment beyond the Mini-Mental State Examination (MMSE) is required and may include cognitive batteries such as the CANTAB (6). Imaging may identify typical patterns of brain volume loss. CSF biomarkers and amyloid positron emission tomography (PET) may demonstrate AD neuropathological change (ADNC) but do not necessarily prove a causal link to the patient's cognitive impairment. Brain biopsies may be obtained at the time of shunt insertion but are therefore only available for those patients who have already been selected for surgery. Our understanding of the role of those biomarkers in the diagnosis of AD has evolved, recognising that they are increasingly prevalent with age and the apoE4 genotype, even in asymptomatic individuals (7). There is an association between CSF biomarkers and outcome (8, 9). While this is not categorical; it seems most robust for Amyloid-β (Aβ)-42 (10). To complicate matters, clinico-pathological relationships in vascular cognitive impairment (VCI) (11) and AD (12) are variable, co-pathology is common, and their interaction on the phenotype remains poorly understood (13).

Typical presentations of Lewy body disease (LBD) (14), progressive supranuclear palsy (PSP) (15) and corticobasal degeneration (CBD) (16) should not pose differential diagnostic difficulties. However, their combination of physical and cognitive decline may be phenotypically similar to iNPH. In their early stages, hallmark clinical features may not have emerged yet and structural brain imaging can be equivocal. Therefore iNPH has to be considered in the differential diagnosis, but co-pathology and mimicry should not be excluded. In this scenario biomarker findings of neurodegenerative disease are either evidence of pathology or co-pathology. If the index of suspicion for iNPH is high, such as after positive tap test, then shunting should be considered. In the shunt responsive iNPH patient neurodegenerative co-pathology seems to modify the clinical phenotype (17). Abnormal DaT imaging is proof of impaired dopaminergic function in the basal ganglia but is aetiologically non-specific and has also been described in iNPH (18, 19). CSF tau species in PSP and CBD have yielded conflicting results (20), CSF RT-QuIC of alpha-synuclein in LBD and tau in PSP and CBD (21) may be more promising.

Little has been published on frontotemporal dementia (FTD) and iNPH. FTD should not cause differential diagnostic problems as difficulties with mobility occur late in the course of the disease. If present early, and typical for iNPH, co-pathology should be suspected. A case report of a C9orf 72 positive patient, with co-occurrence of typical features of FTD and iNPH, described post-shunt improvements in gait and executive tests, while the behavioural disorder remained unaffected (22).

The differential diagnostic assessment of patients suspected of having iNPH, who have early and/or significant cognitive impairment, requires clinical expertise in cognitive and atypical movement disorders to delineate the presenting symptoms and signs. The assessment should conclude with a probabilistic diagnostic statement attributing the findings either to a single (atypical) morbidity or postulating co-morbidity. Neuroimaging, neuropsychology and CSF biomarkers all provide important diagnostic information, emphasising that the best approach to managing patients suspected of having iNPH with significant cognitive impairment is the protocol-driven multidisciplinary team assessment (23).

The existing evidence of cognitive outcomes after shunting has recently been systematically reviewed. Improvement was found in 61% of patients (24). The authors acknowledge several limitations, including a lack of uniform and standardised cognitive outcome measures, and rated the evidence as low to medium.

Probable iNPH patients who also have clinical features or biomarker evidence of CVD (25), AD (26), or LBD (17), if carefully selected for shunt surgery in a tertiary, multidisciplinary setting, have a good chance of improvement, in their gait disorder. They may also experience partial and temporary improvement of their cognitive impairment.

Long-term outcome studies of treated iNPH patients suggest that the numbers developing dementia are significantly greater than in the general population (27, 28). A longitudinal cohort study applying disease modelling to shunted iNPH patients found an overrepresentation of AD compared to the general population after a medium follow up of 5.3 years. Significant predictive factors were cortical biopsy, medial temporal atrophy on MRI and clinical symptoms (29).

Neither aetiology nor pathogenesis of iNPH are well understood (30). The potential role of a loss-of-function variant in CFAP43 (recently described in a Japanese kindred of familial iNPH and confirmed by a knocked out mouse model) in the aetiology of “sporadic” iNPH is currently uncertain (31). Post mortem studies do not go beyond case series and “definite” iNPH, pathological findings remain non-specific (32, 33). There is controversy over whether pathological findings of AD, CVD, LBD and PSP, represent co-morbidity (17), wrong diagnosis (34–36) or even subtype (26). Impaired glymphatic function has been found in AD and iNPH [for a review see (37)] which is probably mediated by aquaporin channels and represents the putative underlying pathophysiology of hydrocephalus and its compensation mechanisms [for a summary see (38)].

Experience from AD, may serve as a model for future research (see Table 1): cooperation between basic science and clinical researchers studying deeply phenotyped, multi-modality assessed and post mortem verified patients has led to increased understanding of aetiology and pathogenesis. These efforts have defined pathological hallmarks and resulted in the development of disease biomarkers. Amyloid PET and CSF amyloid and tau are now available clinically. They have revolutionised clinical treatment trials (39) and are used to screen for ADNC in patients suspected of having iNPH but their role needs to be further clarified. For iNPH, a better understanding of aetiology and pathogenesis, definition of pathological hallmarks and discovery of iNPH specific biomarkers would transform the field. Yet clinical treatment trials in Alzheimer's disease illustrate the challenges of RCT designs in a cognitive disorder. Using multiple modalities and complex sets of cognitive and social outcome measures, they remain in search of the best combination for providing high sensitivity and specificity to reliably demonstrate cognitive change over short time frames (40, 41).

**Table 1:** The pathological basis of cognitive impairment in iNPH and the treatment response to shunting of cognitive impairment in iNPH requires further study.

**Gaps**	**Future research**
Clinico-pathological relationships of cognitive impairment in iNPH not well understood	Longitudinal cohort studies using “deep cognitive phenotyping”, multimodal and novel biomarkers, post-mortem
Treatment: only few high-level studies with advanced cognitive outcome measures controlling for confounding factors (age, education, disease duration, co-morbidities, and cognitive practise effects), cognitive outcome secondary	Multicentre RCTs using composite of detailed cognitive and social outcome measures in addition to multimodal biomarkers

## Imaging

Since the delineation of iNPH as a clinical syndrome, imaging has been needed to demonstrate ventriculomegaly and help evaluate differential diagnoses, including alternative or co-existing causes for cognitive impairment (42). Here, we review the role of imaging in assessing the cognitive aspects of iNPH, focussing on the more recent developments in the evaluation of brain/CSF morphology, diffusion tensor imaging, resting state functional MRI, amyloid PET, and imaging targeting glymphatic clearance.

### Morphology: DESH

In the context of iNPH a widely studied pattern of CSF space morphology is the combination of (i) hydrocephalus (Evans index ≥0.3), (ii) high-convexity/midline tightness and (iii) Sylvian fissure enlargement. This “disproportionately enlarged subarachnoid-space hydrocephalus” (DESH) (43, 44) is found in many but not all (45) cases of iNPH, and as a potential marker of response to shunt surgery (44, 46, 47), some centres have incorporated it into management pathways (30). The pathophysiological mechanisms underlying DESH are unclear but it is likely to be associated with disrupted CSF dynamics (48).

A number of recent studies have now investigated the presence of DESH in the wider population, where it is found in 1–7% and associated with poorer cognition (49–52). A study examining over a thousand participants with either no or only mild cognitive impairment found that DESH was a predictor of progressive cognitive decline independent of established features including age, cortical thickness, or APOE status (48). There is also evidence that in some cases DESH may be a marker of preclinical iNPH: a long term follow up study of asymptomatic individuals with DESH imaging features found that approximately 17% per year subsequently progressed to symptomatic iNPH (53).

### Structural Connectivity: DTI

Diffusion tensor imaging (DTI) is an MRI technique which measures orientation-specific water diffusivity to interrogate brain micro-structure and characterise white matter tracts. White matter injury and dysfunction are proposed components of iNPH pathogenesis and DTI has demonstrated differences in white matter when compared to healthy controls, particularly the corticospinal tract (54) and corpus callosum (55). In several recent studies measures of cognitive impairment in iNPH have been correlated with abnormalities in specific neuroanatomical regions of interest: the forceps minor (56), frontal subcortical white matter (57), right cingulum-hippocampus (58), internal capsules and centrum semiovale (59); these findings are suggestive of the circuits involved in cognitive impairment although study samples are relatively small and patient populations are at risk of other causes of dementia such as AD and VaD. Interestingly, there is evidence that DTI white matter abnormalities can respond to shunt surgery (55, 60) and are potentially partially reversible. Most investigations in iNPH patients have relied on conventional DTI but a few have applied more advanced techniques to further probe tissue microstructure, including kurtosis DTI (57), q-space imaging (61) and neurite orientation dispersion and density imaging (60). There are known technical challenges with comparing DTI datasets between different scanners, however recent experience has confirmed that repeatability and cross-scanner comparability is possible across differing sites (62), allowing future multicentre longitudinal trials.

### Functional Connectivity

Whilst DTI provides measures of structural brain connectivity, MRI techniques are also able to probe functional connectivity. Resting state functional MRI (rsfMRI) examines correlations in brain activity, identifying sets of brain regions that activate simultaneously in the absence of a specific cognitive task. One such set, known as the default mode network (DMN) (63), has been widely studied and changes in DMN connectivity have been associated with cognitive dysfunction across a range of different pathologies (64–66). Altered DMN connectivity has been found in iNPH patients where it is associated with executive dysfunction (67, 68) and poorer cognitive outcomes after shunt placement (68).

Moreover, further studies suggest that the dysfunction seen in iNPH may involve multiple networks in addition to the DMN (69, 70) and can partially normalise after a CSF tap test (70).

### Glymphatic Imaging

There has been increasing interest in imaging targeting the “glymphatic system”: the glia-lymphatic structures which allow the interchange between cerebrospinal fluid and the interstitial space (71–73); this interchange is critical in maintaining interstitial homeostasis and glymphatic dysfunction has been implicated in a range of neurological diseases (74). Multiple *in vivo* MR imaging techniques have been explored (75), particularly those which directly follow the transport of gadolinium based contrast agents (GBCA) after intrathecal administration (76–79). A number of other pilot studies have used MR techniques which do not require an exogeneous tracer: intravoxel incoherent motion MRI (80), DTI (81), chemical exchange saturation transfer imaging (82) and visualisation of lymphatic channels (83).

When applied in iNPH patients, intrathecal GBCA studies have demonstrated differences in CSF redistribution of tracer compared to controls, with significantly more ventricular reflux (77–79)–a finding consistent with previous radionuclide cisternographic studies (84). Interestingly, there also appears to be delayed clearance of GBCA within brain parenchyma (77, 79), including the entorhinal cortex of the mesial temporal lobe (85). It must be noted, however, that the control populations for the above studies were significantly younger than the iNPH group, and increasing age is known to be associated reduced glymphatic function in animal studies (86). Moreover, in the control population the rate of clearance appears to vary widely (77). Further investigation will be required to confirm these findings. The challenges associated with these techniques are well known (75, 87) and, although none are currently suitable for clinical implementation, this is an area of active development.

### Amyloid PET Imaging

Recent work has highlighted the potential of PET imaging for the *in vivo* assessment of amyloid deposition. Amyloid-β (Aβ) is the main component of the plaques found in AD, a frequent co-morbidity in iNPH patients that contributes to cognitive decline (42, 88, 89). Cortical biopsies in iNPH patients frequently detect Aβ, a finding which confers a tenfold increase in the risk of subsequent Alzheimer's disease (90). The PET radiopharmaceutical [^11^C] Pittsburgh Compound B and newer [^18^F]-labelled tracers (flutemetamol, florbetapir, florbetaben) now offer the ability to identify Aβ non-invasively (91–94). Multiple studies have demonstrated strong concordance between histopathology for Aβ and amyloid PET imaging in iNPH patients (91, 95–98), offering a new window on the assessment of this significant pathology.

## Neuropsychology

Individuals with iNPH perform significantly worse than controls on various cognitive measures (6, 99–101). Poorer baseline cognitive status is associated with older age, longer disease duration, worse motor performance (100), and increased mortality after shunt surgery (28). However, variable cognitive patterns have been reported in the literature. Many studies demonstrate early executive dysfunction and psychomotor slowing (102), followed by more widespread cognitive decline at later stages (103). Yet others report early and diffuse cognitive changes, including visuospatial dysfunction and memory impairments (99).

Some studies report post-tap test improvements using either cognitive screens (104) or more comprehensive neuropsychological testing (101). Benefits to cognition have also been reported 3, 12 months (105, 106), and 1–3 years after shunt surgery (107). One meta-analysis (108) reported robust improvements in memory and executive function after shunt surgery. However, post-treatment cognitive outcomes can be variable, and their relationship to other iNPH symptoms remains unclear. Bugalho et al. (99) found no relationship between cognition and gait. Yasar et al. (109) found no improvement in cognitive status after shunt surgery, but an improvement in balance and gait; and Grasso et al. (107) found cognition was not maintained alongside gait improvements at 10 year follow up.

Cognitive screens, such as the MMSE (110), are commonly used in assessment, but may be inadequate for differentiating iNPH from other neurodegenerative disorders (3, 6). Furthermore, practice effects are an important consideration when quantifying true change in cognitive performance across serial assessments (111). Significant practice effects are seen in healthy participants (112) and in post-surgical patients (113) within the first 3 months of serial testing, but were not evident in a sample of iNPH patients over 4 consecutive days (114). It is likely that practice effects differ based on a variety of factors, including age, disease status, test selection, and test-retest interval (111). Interestingly, Duff (111) suggests that practise effects themselves may be important predictors of future cognitive status and treatment outcomes.

Even studies that control for practice effects show variable outcomes. Kambara et al. (115) showed that MMSE scores improved at 3 and 6 months post-surgery, but declined in association with age and poorer scores on an iNPH grading scale. In a large, well-designed study, Solana et al. (116) reported improvements in all cognitive domains 6 months after shunt surgery in group analysis, but only 50% of their participants showed significant improvements on individual analysis.

Another reason for variability in cognitive outcomes may be the presence of alternative or co-morbid neurodegenerative diseases. In one study with a median follow up of 4.8 years, 80% of a shunt responsive group demonstrated cognitive decline, and 46% met the criteria for dementia, with the most common diagnoses being AD and VaD (117). The best predictor of dementia was having memory problems as the first symptom (117). Detailed neuropsychological testing comparing iNPH and Parkinson's disease (PD), in their first year of symptom onset, found more frequent (65% iNPH vs. 25.5% PD) and diffuse cognitive deficits in iNPH (118). Laidet et al. (119) reported that iNPH “mimics” – including PD, atypical PD, VaD, and FTD – failed to demonstrate cognitive improvements after CSF tap test, and that verbal fluency scores distinguished iNPH from this mixed-diagnosis group. Similarly, Liouta et al. (120) used comprehensive neuropsychological tests to show that an iNPH group demonstrated post-tap test and post-shunting cognitive improvements (86 and 97%, respectively), while none of a group including VaD, atypical PD, and FTD showed improvements.

In studies examining neurodegenerative biomarkers, the results are also mixed. A higher incidence of AD biomarkers has been reported in iNPH compared with controls, and was associated with cognitive decline at 2 years (121). While individuals with pathological levels of biomarkers on CSF analysis may show cognitive improvements after tap test (26) and shunt surgery (122, 123), others show less improvements in cognition (122–124). Nerg (6) examined biopsy-acquired AD biomarkers alongside cognition, and found poorer verbal fluency and clock drawing in iNPH, and worse word list learning and picture naming in AD, but little relationship between AD biomarkers and cognitive results.

Specific neuropsychological markers can aid in distinguishing iNPH from other neurodegenerative disorders (6, 117, 125–127), but a discussion of their relative merits is beyond the scope of this review. Therefore, detailed cognitive analysis by a trained neuropsychologist is essential.

### Summary of Neuropsychology

Cognitive impairments in iNPH typically involve executive dysfunction, but may be accompanied by more widespread deficits. Poorer cognition is associated with older age, longer disease duration, co-morbidity, variable outcomes after shunt surgery, and increased mortality.

Variable outcomes may be due to inadequate control for confounding factors, inadequate cognitive measures, or whole-group analyses which average-out individual variability (see Table 1). Where significant cognitive improvements are reported, effect sizes tend to be small, and hence their clinical relevance to individual patients remains uncertain.

Robust neuropsychological methods that control for practice effects in serial testing are needed (128). Detailed cognitive analysis by a trained neuropsychologist is a crucial part of a wider multidisciplinary consensus diagnosis.

## Discussion

We have presented evidence to inform patient management for practitioners confronted with cognitively impaired patients in whom a suspicion of iNPH has been raised. Recent developments have helped to improve differential diagnosis and patient selection for treatment. Neuropsychological differential diagnosis, advanced imaging, and CSF biomarkers are powerful tools starting to enter mainstream clinical use. We encourage active management of these patients through the optimal use of these tools within a structured clinical service. Hence the complex needs of patients with iNPH are best met within a multidisciplinary team. The nosology requires further clarification in prospective cohort studies in cooperation with basic science. An iNPH specific biomarker would revolutionise the field. However, agreement needs to be reached on standardised assessment methods and outcome measures of gait and cognition, where advanced neuropsychological batteries may serve to stratify clinical populations by cognitive features in future RCTs.

## Author Contributions

All authors listed have made a substantial, direct, and intellectual contribution to the work and approved it for publication.

## Conflict of Interest

The authors declare that the research was conducted in the absence of any commercial or financial relationships that could be construed as a potential conflict of interest.

## Publisher's Note

All claims expressed in this article are solely those of the authors and do not necessarily represent those of their affiliated organizations, or those of the publisher, the editors and the reviewers. Any product that may be evaluated in this article, or claim that may be made by its manufacturer, is not guaranteed or endorsed by the publisher.
